# ERICA: prevalence of healthy eating habits among Brazilian adolescents

**DOI:** 10.1590/S01518-8787.2016050006678

**Published:** 2016-02-02

**Authors:** Laura Augusta Barufaldi, Gabriela de Azevedo Abreu, Juliana Souza Oliveira, Debora França dos Santos, Elizabeth Fujimori, Sandra Mary Lima Vasconcelos, Francisco de Assis Guedes de Vasconcelos, Bruno Mendes Tavares

**Affiliations:** I Departamento de Vigilância de Doenças e Agravos Não Transmissíveis e Promoção da Saúde. Secretaria de Vigilância em Saúde. Ministério da Saúde. Brasília, DF, Brasil; II Instituto Medicina Social. Universidade do Estado do Rio de Janeiro. Rio de Janeiro, RJ, Brasil; III Núcleo de Nutrição. Centro Acadêmico de Vitória. Universidade Federal de Pernambuco. Vitória de Santo Antão, PE, Brasil; IV Escola de Enfermagem. Universidade de São Paulo. São Paulo, SP, Brasil; V Faculdade de Nutrição. Universidade Federal de Alagoas. Maceió, AL, Brasil; VI Departamento de Nutrição. Universidade Federal de Santa Catarina. Florianópolis, SC, Brasil; VII Instituto de Saúde e Biotecnologia. Universidade Federal do Amazonas. Coari, AM, Brasil

**Keywords:** Adolescent, Food Habits, Food Preferences, Health Behavior, Cross-Sectional Studies

## Abstract

**OBJECTIVE:**

To describe the prevalence of eating habits considered healthy in adolescents according to sex, age, education level of the mother, school type, session of study, and geographic region.

**METHODS:**

The assessed data come from the Study of Cardiovascular Risks in Adolescents (ERICA), a cross-sectional, national and school-based study. Adolescents of 1,247 schools of 124 Brazilian municipalities were evaluated using a self-administered questionnaire with a section on aspects related to eating behaviors. The following eating behaviors were considered healthy: consuming breakfast, drinking water, and having meals accompanied by parents or legal guardians. All prevalence estimates were presented proportionally, with their respective 95% confidence intervals. The Chi-square test was used to evaluate the differences in healthy eating habits prevalences according to other variables. The module survey of the Stata program version 13.0 was used to analyze complex data.

**RESULTS:**

We evaluated 74,589 adolescents (72.9% of the eligible students). Of these, 55.2% were female, average age being 14.6 years (SD = 1.6). Among Brazilian adolescents, approximately half of them showed healthy eating habits when consuming breakfast, drinking five or more glasses of water a day, and having meals with parents or legal guardians. All analyzed healthy eating habits showed statistically significant differences by sex, age, type of school, session of study, or geographic region **.**

**CONCLUSIONS:**

We suggest that specific actions of intersectoral approach are implemented for the dissemination of the benefits of healthy eating habits. Older female adolescents (15 to 17 years old) who studied in public schools, resided in the Southeast region, and whose mothers had lower education levels, should be the focus of these actions since they present lower frequencies concerning the evaluated healthy habits.

## INTRODUCTION

Adolescence is a period of intense change, in which individuals suffer influences from socioeconomic conditions, family habits, values, and social and cultural rules. Habits and knowledges acquired during this period influence many aspects of adult life related to food, health, and psychosocial development preferences, among others. Thus, healthy eating habits during adolescence are crucial to a healthy productive and reproductive life and for the prevention of noncommunicable chronic diseases in adult life^a^.

For Matias et al.^14^, eating habits comprise a set of actions related to food, starting with the decision, availability, preparation, utensils, eating-schedule and meals division, and finishing with consumption. Having meals with family members, consuming breakfast, and drinking the recommended amount of water are considered healthy habits.

Studies suggest a positive association between having meals with family members and ingesting healthy foods, and inverse association between such habit and obesity occurence^2^ ^,^ ^11^. The National Survey of School Health (PeNSE)^12^, performed with Brazilian students of the ninth grade of elementary school, evaluated these children’s habits of having meals in the presence of their mothers or legal guardians, among other variables of food consumption. Two-thirds of the students frequently had at least one of the main meals in the presence of the mother or legal guardian (for five or more days a week), even though about a quarter of them rarely or had never done this^12^.

According to the Dietary Guidelines for the Brazilian Population, breakfast is one of the three main meals of the day^b^. Compared to snacks, breakfast provides a greater intake of vitamins and minerals and a lower intake of fat and cholesterol^22^. Studies suggest a positive relationship between breakfast consumption and a healthy lifestyle^19^.

Scientific evidences relate the frequent consumption of breakfast to lower risks of overweight and abdominal obesity^16^. On the other hand, the absence of this meal may contribute to calcium deficiency, considering that, generally, breakfast concentrates the highest daily intake of milk and dairy products, sources of the mineral^24^.

Studies conducted in different countries indicate that the proportion of adolescents that consume breakfast ranges from 45.0% to 95.0%^24^. With the increase in the number of individuals living alone and the lack of time to have meals, the way of life of contemporary society have been associated to the decline of breakfast consumption^19^. Proper water intake is vital, preventing dehydration and its adverse effects, such as headaches, urinary lithiasis, and prejudices in cognition, among others^18^. Drinking water before meals and in the place of sugary drinks decrease the consumption of energy and prevent obesity and dental caries^18^ ^,^ ^23^.

Despite the importance of healthy eating habits, studies approaching adolescents are still scarce, which justifies the importance and relevance of this article, whose data represent adolescent students nationally. The aim of this study is to describe the prevalence of healthy eating habits in adolescents according to sex, age, education level of the mother, school type, session of study, and geographic region.

## METHODS

This is an analysis of data from the Study of Cardiovascular Risks in Adolescents (ERICA). The ERICA is a cross-sectional, national and school-based study that aimed to estimate the prevalence of diabetes mellitus, obesity, cardiovascular risk factors, and inflammatory and insulin resistance markers in adolescents aged from 12 to 17 years, enrolled in public and private schools of Brazilian municipalities with more than 100,000 inhabitants.

Data collection took place between March 2013 and December 2014. About 74,589 adolescents of 1,247 schools from 124 Brazilian municipalities were evaluated. The population under research was stratified in 32 strata comprising 27 capitals and five sets of municipalities with more than 100,000 inhabitants in each of the five geographical macro-regions of the Country. For each geographic stratum, schools were selected with probability proportional to size and inversely proportional to the distance from the capital. The sample is representative for medium and large municipalities (> 100,000 inhabitants) at national and regional level, and also for all Brazilian capitals. More details on the sampling process can be found in the publication by Vasconcellos et al.^25^

All students of the selected classes that signed the assent form were interviewed and examined. Adolescents that did not belong to the age group of 12 to 17 years, pregnant girls, and individuals with physical or mental disabilities (temporary or permanent) were considered not eligible and were excluded from the analysis. This study was approved by the Research Ethics Committee of the central coordinating institution of study (IESC/UFRJ) and of each Brazilian State. Bloch et al.^3^ presents details concerning the protocol of this study.

The self-administered questionnaire used was applied with the use of an electronic data collector, the personal digital assistant (PDA). It contained approximately 100 questions divided into 11 sections: sociodemographic aspects, occupational activities, physical practices, eating habits, smoking habits, use of alcoholic beverages, reproductive health, oral health, referred morbidity, sleeping hours, and common mental disorders.

The following eating habits were considered healthy: consuming breakfast, drinking water, and having meals with parents or legal guardians. The section about eating habits included questions on breakfast and on the company of parents or legal guardians during meals as lunch and dinner, with the following answer options: “no”, “sometimes”, “almost every day” and “every day”. For the analysis, we grouped the responses to “almost every day” and “every day”, thus obtaining a variable with the options: “I do not consume it”; “sometimes I consume it” and “I consume it almost/every day”.

We created the variable “meals with parents or legal guardians” by combining the variables “I never have lunch or dinner with my parents or legal guardians” presenting the following answer options: “I never have meals with my parents or legal guardians”; “sometimes I have one or two meals with my parents or legal guardians” and “I often (or always) have at least one meal with my parents or legal guardians”.

Another habit evaluated in the same questionnaire was daily water intake, by considering as answer options: “I do not drink water”, “I drink from one to two glasses”, “I drink from three to four glasses” and “I drink at least five glasses per day”.

The prevalence of healthy habits related to food was analyzed according to the following variables: geographic region (North, Northeast, Midwest, Southeast, and South), sex (male or female), age (in years, analyzed categorically: from 12 and 14, and from 15 to 17), education level of the mother (analyzed categorically: incomplete high school, complete high school or more), session of study (morning or afternoon sessions), and type of school (public or private).

Data analyses were performed in the Strata software, version 13.0, using the module survey for the analysis of complex sample data, in which prevalence estimates were presented proportionally (%), with their respective 95% confidence intervals (95%CI). The Chi-square test was used to evaluate the differences in healthy eating habits prevalences according to other variables.

## RESULTS

Of the 102,327 eligible students, we evaluated about 74,589, which represent 72.9% of the eligible students enrolled in schools and classes previously selected during the sampling process. The study coverage was higher in females (75.8%) than in males (69.6%), and also higher in younger adolescents (77.6%) when compared to those of a higher age group (66.4%). The geographic region where coverage was the highest was the South (81.0%), while the region with the smallest coverage was the Midwest (68.3%). Of the evaluated adolescents, 55.2% were female with average age being 14.6 years (SD = 1.6). Regarding the type of school, 78.7% were public and 71.5% of the students studied in the morning session. About 31.0% of the adolescents evaluated resided in the Northeast, 22.9% in the Southeast, 20.2% in the North, 13.0% in the Midwest, and 12.8% in the South.

More than two-thirds of adolescents (68.0%) “often (or always)” had meals with their parents or legal guardians. However, almost 25.0% had meals in the company of their parents or legal guardians only “sometimes”, while 7.4% declared to “never” do it (Table 1). The prevalence of such habit presented statistically significant difference for all the variables studied, except for study session. We observed higher prevalences of consumption of meals in the presence of parents or legal guardians among younger male adolescents that studied in private schools, resided in the South and Midwest regions, and whose mothers had higher education level (Table 1).

**Table 1:** Prevalence of meals with their parents or legal guardians in Brazilian adolescents aged between 12 and 17 years, considering complex sampling. ERICA, 2013-2014. (N = 74,589)

Variable	Prevalences					

	Never		Sometimes		Often/always	
		
	%	95%CI	%	95%CI	%	95%CI
Sex*						
Female	7.9	7.3-8.5	26.7	25.6-27.8	65.4	64.2-66.6
Male	6.9	6.3-7.6	22.4	21.5-23.5	70.6	69.3-71.9
Age (years)*						
12-14	5.7	5.1-6.4	21.6	20.7-22.5	72.7	71.3-74.0
15-17	9.3	8.6-9.9	27.9	26.8-28.9	62.9	61.6-64.1
Education level of the mother						
						
Incomplete high school	7.5	6.6-8.5	25.5	24.1-27.0	67.0	64.9-69.0
Complete high school or more	7.2	6.4-7.9	22.4	21.2-23.7	70.5	68.8-72.0
Type of school*						
Public	7.5	7.0-8.1	25.3	24.5-26.1	67.2	66.1-68.2
Private	6.7	5.3-8.4	21.1	19.2-23.2	72.2	69.1-75.1
Session of study						
Morning	7.4	6.8-8.1	24.6	23.7-25.7	67.9	66.5-69.3
Afternoon	7.3	6.6-8.1	24.3	22.9-25.8	68.3	66.6-70.0
Geographical region*						
North	6.9	6.4-7.4	25.3	24.3-26.3	67.8	66.7-68.9
Northeast	8.6	7.7-9.5	29.2	27.8-30.6	62.3	60.6-63.9
Midwest	7.0	6.1-8.1	21.2	19.8-22.5	71.8	70.2-73.1
Southeast	7.4	6.6-8.3	24.8	23.5-26.2	67.8	65.9-69.6
South	5.8	4.9-6.7	16.9	15.6-18.2	77.3	75.9-78.6

Total	7.4	6.9-7.9	24.6	23.8-25.3	68.0	67.0-69.0

* Statistically significant difference – Chi-square test.

As for the consumption of breakfast, nearly half of the adolescents (48.5%) reported consuming breakfast frequently (or always), but more than one-fifth (21.9%) usually do not eat this meal (Table 2). We observed a statistically significant difference in the prevalence of breakfast consumption for all the variables analyzed: the greatest prevalences were among younger male adolescents (12 to 14 years), who studied in private schools during the afternoon session, resided in the North and Northeast regions, and whose mothers had higher education (Table 2).

**Table 2:** Prevalence of breakfast consumption in Brazilian adolescents aged between 12 and 17 years, considering complex sampling. ERICA, 2013-2014. (N = 74,589)

Variable	Prevalence					

	Never		Sometimes		Often/Always	
		
	%	95%CI	%	95%CI	%	95%CI
Sex*						
Female	25.8	24.5-27.2	31.8	30.7-33.0	42.3	40.6-44.0
Male	18.2	17.0-19.4	27.2	26.2-28.3	54.6	53.1-56.1
Age (years)*						
12-14	20.1	18.8-21.6	28.3	27.0-29.5	51.6	50.0-53.1
15-17	24.0	22.5-25.7	30.9	29.6-32.3	45.0	42.7-47.4
Education level of the mother						
Incomplete high school	21.9	20.6-23.3	30.3	28.8-31.8	47.8	45.7-49.9
Complete high school or more	20.9	19.8-21.9	27.7	26.4-29.0	51.5	50.1-52.8
Type of school*						
Public	21.9	20.7-23.1	30.9	30.0-31.9	47.2	45.5-48.9
Private	22.5	20.4-24.9	22.8	20.7-25.1	54.6	51.9-57.3
Session of study						
Morning	26.4	25.3-27.7	30.3	29.0-31.7	43.2	41.3-45.1
Afternoon	12.2	11.2-13.3	27.7	26.6-28.9	60.0	58.2-61.8
Geographical region*						
North	13.7	12.6-15.0	25.5	24.2-26.8	60.8	58.6-62.9
Northeast	18.2	16.3-20.2	27.2	25.7-28.6	54.7	52.5-56.8
Midwest	22.3	20.2-24.6	35.2	32.9-37.5	42.6	40.1-45.1
Southeast	23.2	21.4-25.2	30.0	28.5-31.6	46.7	44.2-49.2
South	29.1	26.9-31.4	30.8	28.6-33.0	40.1	36.7-43.6

Total	21.9	20.9-23.1	29.5	28.6-30.4	48.5	47.0-49.9

* Statistically significant difference – Chi-square Test.

Regarding water intake, about half of the adolescents (48.2%) reported drinking five or more glasses of water per day, while 18.9% only drink from one to two glasses, and 1.6% reported not drinking water at all (Table 3). Prevalences of such habit presented significant differences according to sex, age, and geographic region. Younger adolescent boys residing in the Northern region presented the habit of consuming more water daily.

**Table 3:** Prevalence of daily water intake in Brazilian adolescents aged between 12 and 17 years, considering complex sampling. ERICA, 2013-2014. (N = 74,589)

Variable	Prevalence							

	I do not drink water		1 to 2 glasses		3 to 4 glasses		5 or more glasses	
			
	%	95%CI	%	95%CI	%	95%CI	%	95%CI
Sex*								
Female	2.3	1.9-2.6	24.0	22.8-25.3	33.1	31.9-34.2	40.6	39.2-42.0
Male	0.9	0.7-1.0	13.9	12.8-15.0	29.5	28.2-30.8	55.7	53.9-57.5
Age (years)*								
12-14	1.4	1.1-1.6	17.8	16.9-18.6	30.7	29.7-31.7	50.1	48.9-51.4
15-17	1.8	1.5-2.1	20.2	18.9-21.6	31.9	30.6-33.3	46.0	44.2-47.9
Education level of the mother								
Incomplete high school	1.5	1.2-1.8	18.9	17.4-20.5	32.0	30.7-33.3	47.6	45.9-49.5
Complete high school or more	1.4	1.2-1.8	17.2	16.1-18.3	30.9	30.5-33.2	49.5	47.8-51.2
Type of school*								
Public	1.6	1.4-1.8	19.3	18.3-20.3	30.8	29.8-31.8	48.4	47.0-49.7
Private	1.5	0.99-2.2	17.4	15.6-19.4	33.7	31.7-35.8	47.4	44.1-50.7
Session of study								
Morning	1.7	1.4-1.9	19.2	18.2-20.3	31.7	30.6-32.9	47.4	45.9-48.8
Afternoon	1.4	1.1-1.7	18.3	16.9-19.8	30.3	28.8-31.7	50.0	47.8-52.2
Geographical region*								
North	0.3	0.2-0.4	8.9	8.3-9.7	25.1	24.1-26.1	65.7	64.6-66.8
Northeast	0.5	0.3-0.6	12.7	11.2-14.3	29.8	27.5-32.2	57.0	53.7-60.3
Midwest	0.9	0.6-1.2	1.9	14.1-17.3	31.0	29.7-32.4	52.5	50.4-54.6
Southeast	1.8	1.5-2.1	21.2	19.8-22.6	33.1	31.7-34.5	43.9	42.1-45.8
South	4.1	3.2-5.2	30.0	27.8-32.3	30.7	28.9-32.6	35.1	33.5-36.8

Total	1.6	1.4-1.8	18.9	18.1-19.8	31.3	30.4-32.2	48.2	47.0-49.4

* Statistically significant difference – Chi-square test.

## DISCUSSION

Among Brazilian adolescents, approximately half of them showed healthy eating habits when it comes to having meals with their parents or legal guardians, consuming breakfast, and drinking five or more glasses of water per day. However, a significant percentage reported never (or only sometimes) having meals with their parents, never consuming breakfast, and not drinking water or just drinking from one to two glasses daily.

All the analyzed healthy eating habits showed statistically significant differences according to sex, age, type of school, session of study, or geographic region. The results showed that younger male adolescents who studied in private schools, resided in the South and Midwest regions, and whose mothers had higher education, had more meals with their parents or legal guardians. We observed that the greatest prevalences of breakfast consumption, by its turn, were among younger male adolescents who studied in private schools, during the afternoon session, resided in the North and Northeast regions of Brazil, and whose mothers had higher education level. Water intake was higher among younger male adolescents who resided in the North region.

Having family meals is an important aspect of the familiar environment that promotes healthy eating habits in adolescence and its maintenance in adulthood. It also contributes to the reduction of unhealthy eating practices, influencing positively increased consumption of fruits, vegetables, and dairy products and decreased consumption of sugary drinks^7^. Despite not having a consensus on the subject, some records consider family meals as contributors to the prevention of obesity. Results of the meta-analyses showed that children and adolescents that have regular family meals were less likely to be overweight^7^. In addition to healthy eating habits and obesity prevention, family meals can help to reduce violence, sexual activity, mental health problems, and self-injury among children and adolescents^6^ ^,^ ^21^. They can also promote positive family interactions, including greater communication, socialization, and transmission of values and culture^10^. The frequency of meals with parents or legal guardians observed in this study (68.0%) is similar to that found by PeNSE^12^, which found that 62.6% of adolescents had (at least) lunch or dinner with the mother or legal guardian. The association with the variable sex also showed to be similar to the results from PeNSE, which observed a higher prevalence of this habit among males, but without distinction between public and private school students^12^, differently to what we observed in this study.

The greatest prevalence of breakfast by male adolescents reaffirms the results found in other studies^9^ ^,^ ^20^. To Keski-Rahkonen et al.^9^, the smallest prevalence among girls may be due to body dissatisfaction and the attempt to lose weight; these authors found associations between having breakfast and having breakfast accompanied by the parents and not having this meal with low education levels at age 16, smoking habits, frequent use of alcohol beverages, little exercise and high body mass indices.

The inverse relation we found in this study between breakfast and age is similar to the observed in other studies. Breakfast consumption seems to increase with age when it comes to adults (between 18 and 60 years)^4^ and decrease with age in children and adolescents (between four and 18 years)^19^. The main excuses that adolescents who do not have breakfast give are: “lack of time”, “lack of hunger”, “intention of dieting” and “preference for sleeping”^24^. Adolescents from private schools and children of mothers with higher education (complete high school or more) consume breakfast and meals with parents or legal guardians with greater frequency. This indicates that socioeconomic status may be related to these eating habits, i.e., adolescents from higher socioeconomic levels present healthier behaviors.

Although water balance involves complex mechanisms, an adequate supply of water represents a health practice as important as the adoption of healthy eating (being also considered part of it)^8^. Moreover, drinking water can help controlling weight^15^ ^,^ ^23^ and the consumption of sugary drinks; consequently, it prevents problems associated with the consumption of these drinks, such as dental caries^1^, obesity^5^, and type 2 diabetes^13^. Park et al.^17^ observed that 54.0% of high school students from the United States reported drinking water less than three times a day. The authors also found a significant association between low intakes of pure water and the following factors: lower consumption of milk, fruits (including 100% fruit juices), and vegetables; higher consumption of soft drinks, other sugary drinks, and fast food; and physical inactivity. Despite quantifying water intake differently, the results of this study suggest that Brazilian adolescents present a healthier level of water intake than American adolescents.

Considering the size of the sample and the representativeness of the study, ERICA brings important contributions to the mapping of healthy eating habits among adolescents. However, for being a cross-sectional study, it makes possible extrapolations on causal factors.

Whereas adolescence represents a chance for the prevention of chronic diseases related to nutrition in adulthood^a^, the results of this study suggest that specific actions of intersectoral approach are necessary. They should be implemented to spread the benefits of having meals with the family, consuming all meals (especially breakfast), and drinking water, thus building healthy eating habits that tend to remain during adulthood. Older female adolescents (15 to 17 years) who studied in public schools, resided in the Southeast region, whose mothers had lower education levels, should be the focus of these actions since they present lower frequencies concerning the evaluated healthy habits. We recommend future investigations to assess the relationship between healthy eating habits employing anthropometric, biochemical, and food consumption data.

## Footnotes

a World Health Organization. Nutrition in adolescence: issues and challenges for the health sector: issues in adolescent health and development. Geneva; 2005 [cited 2015 Jul 31]. (WHO discussion paper on adolescence). Available from: http://apps.who.int/iris/bitstream/10665/43342/1/9241593660_eng.pdf

b Ministério da Saúde, Secretaria de Atenção à Saúde, Departamento de Atenção Básica. Guia alimentar para a população brasileira. 2.ed. Brasília (DF); 2014.
