# Supplementary material for: ERICA: prevalence of healthy eating habits among Brazilian adolescents
Source: Rev Saude Publica. 2016 Feb 2;50(Suppl 1):6s. doi: 10.1590/S01518-8787.2016050006678 (PMC4766945; doi:10.1590/S01518-8787.2016050006678)
Supplement: Translation

# Translation: ERICA: prevalência de comportamentos alimentares saudáveis em adolescentes brasileiros

**Authors:** Laura Augusta Barufaldi, Gabriela de Azevedo Abreu, Juliana Souza Oliveira, Debora França dos Santos, Elizabeth Fujimori, Sandra Mary Lima Vasconcelos, Francisco de Assis Guedes de Vasconcelos, Bruno Mendes Tavares

**Affiliations:** I Departamento de Vigilância de Doenças e Agravos Não Transmissíveis e Promoção da Saúde. Secretaria de Vigilância em Saúde. Ministério da Saúde. Brasília, DF, Brasil; II Instituto Medicina Social. Universidade do Estado do Rio de Janeiro. Rio de Janeiro, RJ, Brasil; III Núcleo de Nutrição. Centro Acadêmico de Vitória. Universidade Federal de Pernambuco. Vitória de Santo Antão, PE, Brasil; IV Escola de Enfermagem. Universidade de São Paulo. São Paulo, SP, Brasil; V Faculdade de Nutrição. Universidade Federal de Alagoas. Maceió, AL, Brasil; VI Departamento de Nutrição. Universidade Federal de Santa Catarina. Florianópolis, SC, Brasil; VII Instituto de Saúde e Biotecnologia. Universidade Federal do Amazonas. Coari, AM, Brasil

**Keywords:** Adolescente, Hábitos Alimentares, Preferências Alimentares, Comportamentos Saudáveis, Estudos Transversais

## Abstract

**OBJETIVO:**

Descrever a prevalência de comportamentos alimentares considerados saudáveis em adolescentes brasileiros, segundo sexo, idade, escolaridade da mãe, tipo de escola, turno de estudo e região geográfica.

**MÉTODOS:**

Os dados analisados provêm do Estudo de Riscos Cardiovasculares em Adolescentes (ERICA), estudo transversal, nacional e de base escolar. Foram avaliados adolescentes de 1.247 escolas em 124 municípios brasileiros, utilizando questionário autopreenchível que incluía um bloco sobre aspectos relacionados ao comportamento alimentar. Foram considerados saudáveis os seguintes comportamentos alimentares: consumo de café da manhã, ingestão de água e realização de refeições com os pais ou responsáveis. As estimativas das prevalências foram apresentadas em proporções, com seus respectivos intervalos de confiança de 95%. O teste Qui-quadrado foi utilizado para avaliar a diferença das prevalências dos comportamentos alimentares saudáveis de acordo com as demais variáveis. Utilizou-se o módulo *survey* do programa Stata versão 13,0 para análise de dados de amostra complexa.

**RESULTADOS:**

Foram avaliados 74.589 adolescentes (72,9% dos alunos elegíveis). Desses, 55,2% eram do sexo feminino e a média de idade foi de 14,6 anos (DP = 1,6). Entre os adolescentes brasileiros, aproximadamente metade apresentou comportamentos alimentares saudáveis quanto ao consumo de café da manhã, realização de refeições com os pais ou responsáveis e ingestão de cinco ou mais copos de água por dia. Todos os comportamentos alimentares saudáveis analisados apresentaram diferença estatisticamente significativa por sexo, idade, tipo de escola, turno de estudo ou região geográfica.

**CONCLUSÕES:**

Sugere-se que ações específicas, de abordagem intersetorial, sejam implementadas para a disseminação dos benefícios dos comportamentos alimentares saudáveis. Adolescentes do sexo feminino, mais velhos (15 a 17 anos), cujas mães têm escolaridade mais baixa, alunos de escolas públicas e da região Sudeste, deveriam ser o foco dessas ações já que são os que apresentam menores frequências dos comportamentos saudáveis analisados.

## INTRODUÇÃO

A adolescência é um período de intensas mudanças, no qual os indivíduos sofrem influências de condições socioeconômicas, hábitos familiares, valores e regras sociais e culturais. Hábitos e conhecimentos adquiridos nesse período têm influência sobre muitos aspectos da vida adulta relacionados à alimentação, saúde, preferências e desenvolvimento psicossocial, entre outros. Assim, comportamentos alimentares saudáveis durante a adolescência são fundamentais para uma vida produtiva e reprodutiva saudável e para a prevenção de doenças crônicas não transmissíveis na vida adulta^a^.

Para Matias et al.^14^, o comportamento alimentar é um conjunto de ações relacionadas ao alimento, que começa com a decisão, disponibilidade, modo de preparo, utensílios, horários e divisão das refeições, e encerra com a ingestão. Alguns comportamentos alimentares são considerados saudáveis, tais como realizar refeições com a família, consumir café da manhã e ingerir a quantidade recomendada de água.

Estudos sugerem associação positiva entre realizar refeições com a família e ingerir alimentos saudáveis e associação inversa entre este comportamento e a ocorrência de obesidade^2^ ^,^ ^11^. A Pesquisa Nacional de Saúde do Escolar (PeNSE)^12^, realizada com escolares brasileiros do nono ano do ensino fundamental, avaliou o hábito de realizar refeições na presença da mãe ou responsável, entre outras variáveis de consumo alimentar. Dois terços dos estudantes realizava frequentemente (cinco ou mais dias na semana) pelo menos uma das principais refeições na presença da mãe ou responsável, embora cerca de um quarto deles raramente ou nunca tivessem essa prática^12^.

Segundo o Guia Alimentar para a População Brasileira, o café da manhã é uma das três principais refeições do dia^b^. Comparada aos lanches, a refeição matinal proporciona maior ingestão de vitaminas e minerais e menor ingestão de gorduras e colesterol^22^. Estudos sugerem relação positiva entre o consumo de café da manhã e estilo de vida saudável^19^.

Evidências científicas relacionam o consumo frequente de café da manhã com baixo risco de sobrepeso e obesidade abdominal^16^. Por outro lado, ausência dessa refeição pode favorecer deficiência de cálcio, uma vez que, geralmente, é a refeição que concentra o maior consumo diário de leite e derivados, fontes desse mineral^24^.

Estudos realizados em diferentes países indicam que a proporção de adolescentes que consome o café da manhã varia de 45,0% a 95,0%^24^. O estilo de vida da sociedade contemporânea, com aumento do número de indivíduos que moram sozinhos e falta de tempo para realizar as refeições, tem sido associado ao declínio de consumo do café da manhã^19^.

A ingestão adequada de água é vital, prevenindo a desidratação e seus efeitos adversos, como cefaléia, litíase urinária e prejuízo da cognição, entre outros^18^. A substituição de bebidas açucaradas por água e o seu consumo antes das refeições associam-se a menor consumo de energia com prevenção da obesidade e também de cáries dentárias^18^ ^,^ ^23^.

Apesar da importância dos comportamentos alimentares saudáveis, ainda são escassos os estudos realizados em adolescentes, o que justifica a relevância e pertinência do presente artigo, cujos dados têm representatividade nacional de adolescentes escolares. O objetivo deste estudo foi descrever a prevalência de comportamentos alimentares considerados saudáveis em adolescentes brasileiros, segundo sexo, idade, escolaridade da mãe, tipo de escola, turno de estudo e região geográfica.

## MÉTODOS

Trata-se da análise de dados provenientes do Estudo de Riscos Cardiovasculares em Adolescentes (ERICA). O ERICA é um estudo transversal, nacional e de base escolar que teve como objetivo estimar a prevalência de diabetes mellitus, obesidade, fatores de risco cardiovascular e de marcadores inflamatórios e de resistência à insulina em adolescentes de 12 a 17 anos, matriculados em escolas públicas e privadas, de cidades brasileiras com mais de 100 mil habitantes.

A coleta de dados ocorreu entre março de 2013 e dezembro de 2014. Foram avaliados 74.589 adolescentes de 1.247 escolas em 124 municípios brasileiros. A população da pesquisa foi estratificada em 32 estratos constituídos por 27 capitais e cinco conjuntos de municípios com mais de 100 mil habitantes em cada uma das cinco macrorregiões geográficas do País. Para cada estrato geográfico, as escolas foram selecionadas com probabilidade proporcional ao tamanho e inversamente proporcional à distância da capital. A amostra é representativa para municípios de médio e grande porte (> 100 mil habitantes) em âmbito nacional, regional e para as capitais brasileiras. Mais detalhes sobre o processo de amostragem encontram-se na publicação de Vasconcellos et al.^25^

Foram entrevistados e examinados todos os alunos das turmas selecionadas que assinaram o termo de assentimento. Foram excluídos das análises, por não serem considerados elegíveis, os adolescentes que não pertenciam à faixa etária de 12 a 17 anos, adolescentes grávidas e os indivíduos com deficiência física ou mental, temporária ou permanente. O estudo foi aprovado pelos Comitês de Ética em Pesquisa da instituição da coordenação central do estudo (IESC/UFRJ) e de cada estado brasileiro. Detalhes sobre o protocolo do estudo encontram-se em Bloch et al.^3^

O questionário utilizado foi autopreenchível e aplicado com o uso de um coletor eletrônico de dados, o *personal digital assistant* (PDA). Continha cerca de 100 questões distribuídas em 11 blocos: aspectos sociodemográficos, atividades ocupacionais, atividade física, comportamento alimentar, tabagismo, uso de bebidas alcoólicas, saúde reprodutiva, saúde bucal, morbidade referida, duração do sono e transtorno mental comum.

Foram considerados saudáveis os seguintes comportamentos alimentares: consumo de café da manhã, ingestão de água e realização de refeições com os pais ou responsáveis. O bloco de comportamento alimentar incluiu perguntas sobre a realização do café da manhã e a companhia dos pais ou responsáveis nas refeições (almoço e jantar), com as seguintes opções de resposta: “não”, “às vezes”, “quase todos os dias” e “todos os dias”. Para a análise, as respostas “quase todos os dias” e “todos os dias” foram agrupadas, obtendo-se uma variável com as opções: “não consome”; “consome às vezes” e “consome quase/todos os dias”.

A variável refeição na companhia dos pais ou responsáveis foi criada a partir da combinação das variáveis “almoço e jantar com pais ou responsáveis” de forma que permanecessem as seguintes opções: “nunca faz refeições com os pais ou responsáveis”; “às vezes faz uma ou duas refeições com pais ou responsáveis” e “quase sempre ou sempre faz pelo menos uma das refeições com os pais ou responsáveis”.

Outro comportamento avaliado no mesmo questionário foi o consumo diário de água, sendo consideradas como opções de resposta: “não bebe água”, “bebe um a dois copos”, “bebe três a quatro copos” e “bebe pelo menos cinco copos por dia”.

A prevalência dos comportamentos saudáveis relacionados à alimentação foi analisada de acordo com as variáveis: região geográfica (Norte, Nordeste, Centro-Oeste, Sudeste e Sul) sexo (masculino ou feminino), idade (em anos, analisada de forma categorizada: de 12 a 14 e de 15 a 17), escolaridade da mãe (analisada de forma categorizada: até ensino médio incompleto e ensino médio completo ou mais), turno de estudo (manhã ou tarde) e tipo de escola (pública ou privada).

As análises dos dados foram realizadas no *software* Stata, versão 13.0, utilizando-se o módulo *survey* para análise de dados de amostra complexa, onde as estimativas das prevalências foram apresentadas em proporções (%), com seus respectivos intervalos de confiança de 95% (IC95%). O teste Qui-quadrado foi utilizado para avaliar a diferença das prevalências dos comportamentos alimentares saudáveis de acordo com as demais variáveis.

## RESULTADOS

Dos 102.327 alunos elegíveis, foram avaliados 74.589, que representam 72,9% dos alunos elegíveis matriculados nas escolas e turmas selecionadas no processo de amostragem. Essa cobertura foi maior no sexo feminino (75,8%) do que no sexo masculino (69,6%), e maior também nos adolescentes mais jovens (77,6%) quando comparado aos de faixa etária mais alta (66,4%). A região geográfica onde a cobertura foi mais elevada foi a Sul (81,0%) e a região com menor cobertura foi a Centro-Oeste (68,3%). Dos adolescentes avaliados, 55,2% eram do sexo feminino e a média de idade foi de 14,6 anos (DP = 1,6). Em relação ao tipo de escola, 78,7% eram públicas, e 71,5% dos alunos estudavam no turno da manhã. Dos adolescentes avaliados, 31,0% eram do Nordeste, 22,9% do Sudeste, 20,2% do Norte, 13,0% do Centro-Oeste e 12,8% da região Sul.

Mais de dois terços dos adolescentes (68,0%) “sempre ou quase sempre” realizavam refeições com os pais ou responsáveis. Entretanto, quase 25,0% realizava refeições na companhia dos pais ou responsáveis apenas “às vezes” e 7,4%, “nunca” (Tabela 1). A prevalência desse comportamento apresentou diferença estatisticamente significativa para todas as variáveis estudadas, exceto em relação ao turno de estudo. Maior prevalência de consumo de refeições com os pais ou responsáveis foi observada entre adolescentes do sexo masculino, mais jovens, cujas mães tinham maior escolaridade, eram de escolas privadas e residiam nas regiões Sul e Centro-Oeste (Tabela 1).

**Tabela 1:** Prevalência de realização de refeições com os pais ou responsáveis nos adolescentes brasileiros de 12 a 17 anos, considerando amostragem complexa. ERICA, 2013-2014. (N = 74.589)

Variável	Prevalência					

	Nunca		Às vezes		Quase sempre/Sempre	
		
	%	IC95%	%	IC95%	%	IC95%
Sexo*						
Feminino	7,9	7,3-8,5	26,7	25,6-27,8	65,4	64,2-66,6
Masculino	6,9	6,3-7,6	22,4	21,5-23,5	70,6	69,3-71,9
Idade (anos)*						
12-14	5,7	5,1-6,4	21,6	20,7-22,5	72,7	71,3-74,0
15-17	9,3	8,6-9,9	27,9	26,8-28,9	62,9	61,6-64,1
Escolaridade da mãe*						
Até ensino médio incompleto	7,5	6,6-8,5	25,5	24,1-27,0	67,0	64,9-69,0
Ensino médio completo ou mais	7,2	6,4-7,9	22,4	21,2-23,7	70,5	68,8-72,0
Tipo de escola*						
Pública	7,5	7,0-8,1	25,3	24,5-26,1	67,2	66,1-68,2
Privada	6,7	5,3-8,4	21,1	19,2-23,2	72,2	69,1-75,1
Turno de estudo						
Manhã	7,4	6,8-8,1	24,6	23,7-25,7	67,9	66,5-69,3
Tarde	7,3	6,6-8,1	24,3	22,9-25,8	68,3	66,6-70,0
Região geográfica*						
Norte	6,9	6,4-7,4	25,3	24,3-26,3	67,8	66,7-68,9
Nordeste	8,6	7,7-9,5	29,2	27,8-30,6	62,3	60,6-63,9
Centro-Oeste	7,0	6,1-8,1	21,2	19,8-22,5	71,8	70,2-73,1
Sudeste	7,4	6,6-8,3	24,8	23,5-26,2	67,8	65,9-69,6
Sul	5,8	4,9-6,7	16,9	15,6-18,2	77,3	75,9-78,6

Total	7,4	6,9-7,9	24,6	23,8-25,3	68,0	67,0-69,0

* Diferença estatisticamente significativa – Teste Qui-quadrado.

Quanto ao consumo do café da manhã, quase metade dos adolescentes (48,5%) relatou consumir café da manhã quase sempre ou sempre, porém mais de um quinto (21,9%) não realizavam essa refeição (Tabela 2). Observou-se diferença estatisticamente significativa nas prevalências de consumo do café da manhã para todas as variáveis analisadas: maiores prevalências foram observadas nos adolescentes do sexo masculino, entre os mais jovens (12 a 14 anos), cujas mães apresentavam maior escolaridade, que estudavam em escolas privadas, no turno da tarde e residentes nas regiões Norte e Nordeste (Tabela 2).

**Tabela 2:** Prevalência de consumo de café da manhã nos adolescentes brasileiros de 12 a 17 anos, considerando amostragem complexa. ERICA, 2013-2014. (N = 74.589)

Variável	Prevalência					

	Nunca		Às vezes		Quase sempre/Sempre	
		
	%	IC95%	%	IC95%	%	IC95%
Sexo*						
Feminino	25,8	24,5-27,2	31,8	30,7-33,0	42,3	40,6-44,0
Masculino	18,2	17,0-19,4	27,2	26,2-28,3	54,6	53,1-56,1
Idade (anos)*						
12-14 anos	20,1	18,8-21,6	28,3	27,0-29,5	51,6	50,0-53,1
15-17 anos	24,0	22,5-25,7	30,9	29,6-32,3	45,0	42,7-47,4
Escolaridade da mãe*						
Até ensino médio incompleto	21,9	20,6-23,3	30,3	28,8-31,8	47,8	45,7-49,9
Ensino médio completo ou mais	20,9	19,8-21,9	27,7	26,4-29,0	51,5	50,1-52,8
Tipo de escola*						
Pública	21,9	20,7-23,1	30,9	30,0-31,9	47,2	45,5-48,9
Privada	22,5	20,4-24,9	22,8	20,7-25,1	54,6	51,9-57,3
Turno de estudo*						
Manhã	26,4	25,3-27,7	30,3	29,0-31,7	43,2	41,3-45,1
Tarde	12,2	11,2-13,3	27,7	26,6-28,9	60,0	58,2-61,8
Região geográfica*						
Norte	13,7	12,6-15,0	25,5	24,2-26,8	60,8	58,6-62,9
Nordeste	18,2	16,3-20,2	27,2	25,7-28,6	54,7	52,5-56,8
Centro-Oeste	22,3	20,2-24,6	35,2	32,9-37,5	42,6	40,1-45,1
Sudeste	23,2	21,4-25,2	30,0	28,5-31,6	46,7	44,2-49,2
Sul	29,1	26,9-31,4	30,8	28,6-33,0	40,1	36,7-43,6

Total	21,9	20,9-23,1	29,5	28,6-30,4	48,5	47,0-49,9

* Diferença estatisticamente significativa – Teste Qui-quadrado.

Com relação ao consumo de água, cerca de metade dos adolescentes (48,2%) referiu ingerir cinco ou mais copos de água por dia, enquanto 18,9% consumiam apenas um a dois copos e 1,6% relatou não consumir água (Tabela 3). As prevalências desse comportamento apresentaram diferenças significativas segundo sexo, idade e região geográfica. Meninos, mais jovens e adolescentes residentes na região Norte apresentaram hábito de consumir mais água diariamente.

**Tabela 3:** Prevalência do consumo diário de água nos adolescentes brasileiros de 12 a 17 anos, considerando amostragem complexa. ERICA, 2013-2014. (N = 74.589)

Variável	Prevalência							

	Não bebe		1 a 2 copos		3 a 4 copos		5 ou mais copos	
			
	%	IC95%	%	IC95%	%	IC95%	%	IC95%
Sexo*								
Feminino	2,3	1,9-2,6	24,0	22,8-25,3	33,1	31,9-34,2	40,6	39,2-42,0
Masculino	0,9	0,7-1,0	13,9	12,8-15,0	29,5	28,2-30,8	55,7	53,9-57,5
Idade (anos)*								
12-14 anos	1,4	1,1-1,6	17,8	16,9-18,6	30,7	29,7-31,7	50,1	48,9-51,4
15-17 anos	1,8	1,5-2,1	20,2	18,9-21,6	31,9	30,6-33,3	46,0	44,2-47,9
Escolaridade da mãe								
Até ensino médio incompleto	1,5	1,2-1,8	18,9	17,4-20,5	32,0	30,7-33,3	47,6	45,9-49,5
Ensino médio completo ou mais	1,4	1,2-1,8	17,2	16,1-18,3	30,9	30,5-33,2	49,5	47,8-51,2
Tipo de escola								
Pública	1,6	1,4-1,8	19,3	18,3-20,3	30,8	29,8-31,8	48,4	47,0-49,7
Privada	1,5	0,99-2,2	17,4	15,6-19,4	33,7	31,7-35,8	47,4	44,1-50,7
Turno de estudo								
Manhã	1,7	1,4-1,9	19,2	18,2-20,3	31,7	30,6-32,9	47,4	45,9-48,8
Tarde	1,4	1,1-1,7	18,3	16,9-19,8	30,3	28,8-31,7	50,0	47,8-52,2
Região geográfica*								
Norte	0,3	0,2-0,4	8,9	8,3-9,7	25,1	24,1-26,1	65,7	64,6-66,8
Nordeste	0,5	0,3-0,6	12,7	11,2-14,3	29,8	27,5-32,2	57,0	53,7-60,3
Centro-Oeste	0,9	0,6-1,2	1,9	14,1-17,3	31,0	29,7-32,4	52,5	50,4-54,6
Sudeste	1,8	1,5-2,1	21,2	19,8-22,6	33,1	31,7-34,5	43,9	42,1-45,8
Sul	4,1	3,2-5,2	30,0	27,8-32,3	30,7	28,9-32,6	35,1	33,5-36,8

Total	1,6	1,4-1,8	18,9	18,1-19,8	31,3	30,4-32,2	48,2	47,0-49,4

* Diferença estatisticamente significativa – Teste Qui-quadrado.

## DISCUSSÃO

Entre os adolescentes brasileiros, aproximadamente metade apresentou comportamentos alimentares saudáveis no que se refere à realização de refeições com os pais ou responsáveis, consumo de café da manhã e ingestão de cinco ou mais copos de água por dia. Entretanto, um percentual importante declarou nunca ou apenas às vezes realizar as refeições com os pais, nunca consumir café da manhã, e não beber ou ingerir apenas um a dois copos de água diariamente.

Todos os comportamentos alimentares saudáveis analisados apresentaram diferença estatisticamente significativa segundo sexo, idade, tipo de escola, turno de estudo ou região geográfica. Os resultados mostraram que realizam mais refeições com pais ou responsáveis, adolescentes do sexo masculino, mais jovens, cujas mães apresentam escolaridade mais alta, alunos de escola privada e residentes nas regiões Sul e Centro-Oeste. As maiores prevalências de consumo do café da manhã, por sua vez, foram observadas nos adolescentes do sexo masculino, mais jovens, cujas mães apresentam escolaridade mais alta, que estudam em escolas privadas, no turno da tarde e que residem nas regiões Norte e Nordeste do Brasil. Já o consumo de água foi maior entre os meninos, mais jovens e residentes na região Norte.

A realização de refeições em família é um importante aspecto do ambiente familiar que influencia a promoção de comportamentos alimentares saudáveis na adolescência e sua manutenção na fase adulta. Contribui para a redução das práticas de alimentação pouco saudáveis, com influência positiva para o maior consumo de frutas, vegetais e produtos lácteos e menor consumo de bebidas açucaradas^7^. Embora não exista consenso, há registros de que as refeições em família também contribuem para a prevenção de obesidade. Resultados de meta-análise evidenciaram que crianças e adolescentes com refeições regulares em família apresentavam menor probabilidade de ter sobrepeso^7^. Além de comportamentos alimentares saudáveis e prevenção da obesidade, as refeições familiares podem contribuir para a redução da violência, atividade sexual, problemas de saúde mental e auto-agressão entre crianças e adolescentes^6^ ^,^ ^21^. Também promovem interações familiares positivas, incluindo maior comunicação, socialização e transmissão de valores e cultura^10^. A frequência de refeições realizadas com os pais ou responsáveis obtida neste estudo (68,0%) é similar à encontrada pela PeNSE^12^, que constatou que 62,6% dos adolescentes realizava pelo menos o almoço ou o jantar com a mãe ou responsável. A associação desse comportamento com a variável sexo também se mostrou semelhante aos resultados da PeNSE, que observou maior prevalência desse hábito no sexo masculino, porém sem distinção entre alunos de escolas públicas e privadas^12^, diferentemente do verificado no presente estudo.

A maior prevalência de realização do café da manhã por adolescentes do sexo masculino reitera resultados de outros estudos^9^ ^,^ ^20^. Para Keski-Rahkonen et al.^9^, a menor prevalência nas meninas pode estar associada à insatisfação corporal e à tentativa de perder peso; esses autores encontraram associação entre realizar café da manhã e consumo de café da manhã pelos pais, e não realizar essa refeição e baixo nível de escolaridade aos 16 anos, tabagismo, uso frequente de álcool, pouco exercício físico e alto índice de massa corporal.

A relação inversa, encontrada neste estudo, entre café da manhã e idade assemelha-se à observada em outras investigações. O consumo do café da manhã parece aumentar com a idade quando se trata de adultos (entre 18 e 60 anos)^4^ e diminuir com a idade em crianças e adolescentes (entre 4 e 18 anos)^19^. As principais justificativas dos adolescentes que não tomam café da manhã são “falta de tempo”, “falta de fome”, “intenção de fazer dieta” e “preferência por dormir”^24^. Os adolescentes de escolas privadas e filhos de mães com escolaridade mais alta (ensino médio completo ou mais) consomem o café da manhã e realizam refeições com pais ou responsáveis com maior frequência. Isso indica que o nível socioeconômico pode estar relacionado com esses comportamentos alimentares, isto é, adolescentes de níveis socioeconômico mais alto apresentam comportamentos mais saudáveis.

Embora o balanço hídrico envolva mecanismos complexos, o aporte adequado de água representa uma prática de saúde tão importante quanto a adoção de uma alimentação saudável, sendo inclusive parte dela^8^. Além disso, consumir água pode ajudar no controle de peso^15^ ^,^ ^23^ e do consumo de bebidas açucaradas; consequentemente, previne problemas associados com o consumo dessas bebidas, como cárie dentária^1^, obesidade^5^ e diabetes tipo 2^13^. Park et al.^17^ observaram que 54,0% dos estudantes do ensino médio nos Estados Unidos relataram beber água menos de três vezes por dia. Os autores também encontraram associação significativa entre baixa ingestão de água pura e os seguintes fatores: menor consumo de leite, frutas (incluindo suco 100% da fruta) e legumes; maior consumo de refrigerantes, outras bebidas açucaradas e *fast-food*; e inatividade física. Apesar de quantificar de forma diferente o consumo de água, os resultados do presente estudo sugerem que os adolescentes brasileiros apresentam um consumo de água mais saudável que os adolescentes americanos.

Considerando o tamanho amostral e a representatividade do estudo, o ERICA traz importante contribuição para o mapeamento dos comportamentos alimentares saudáveis nos adolescentes brasileiros. Entretanto, por possuir delineamento transversal, não possibilita extrapolações sobre fatores causais.

Considerando que a adolescência representa uma oportunidade para a prevenção de doenças crônicas relacionadas à nutrição na vida adulta^a^, os resultados deste estudo sugerem que ações específicas, de abordagem intersetorial, são necessárias. Devem ser implementadas visando à disseminação dos benefícios da realização de refeições com a família, da prática de se consumir todas as refeições, especialmente o café da manhã, e do hábito de ingerir água, consolidando comportamentos alimentares saudáveis que tendem a se manter durante a vida adulta. De acordo com os resultados deste estudo, adolescentes do sexo feminino, mais velhos (15 a 17 anos), cujas mães apresentam menor escolaridade, alunos de escolas públicas e da região Sudeste deveriam ser o foco dessas ações, já que são os que apresentam menores frequências dos comportamentos saudáveis analisados. Recomenda-se que investigações futuras avaliem as relações entre os comportamentos alimentares saudáveis com dados antropométricos, bioquímicos e de consumo alimentar.

## Footnotes

a World Health Organization. Nutrition in adolescence: issues and challenges for the health sector: issues in adolescent health and development. Geneva; 2005 [citado 2015 jul 31]. (WHO discussion paper on adolescence). Disponível em: http://apps.who.int/iris/bitstream/10665/ 43342/1/9241593660_eng.pdf

b Ministério da Saúde, Secretaria de Atenção à Saúde, Departamento de Atenção Básica. Guia alimentar para a população brasileira. 2.ed. Brasília (DF); 2014.
